# Supplementary figures and images for: QTL mapping of seedling and field resistance to stem rust in DAKIYE/Reichenbachii durum wheat population
Source: PLoS One. 2022 Oct 6;17(10):e0273993. doi: 10.1371/journal.pone.0273993 (PMC9536579; doi:10.1371/journal.pone.0273993)

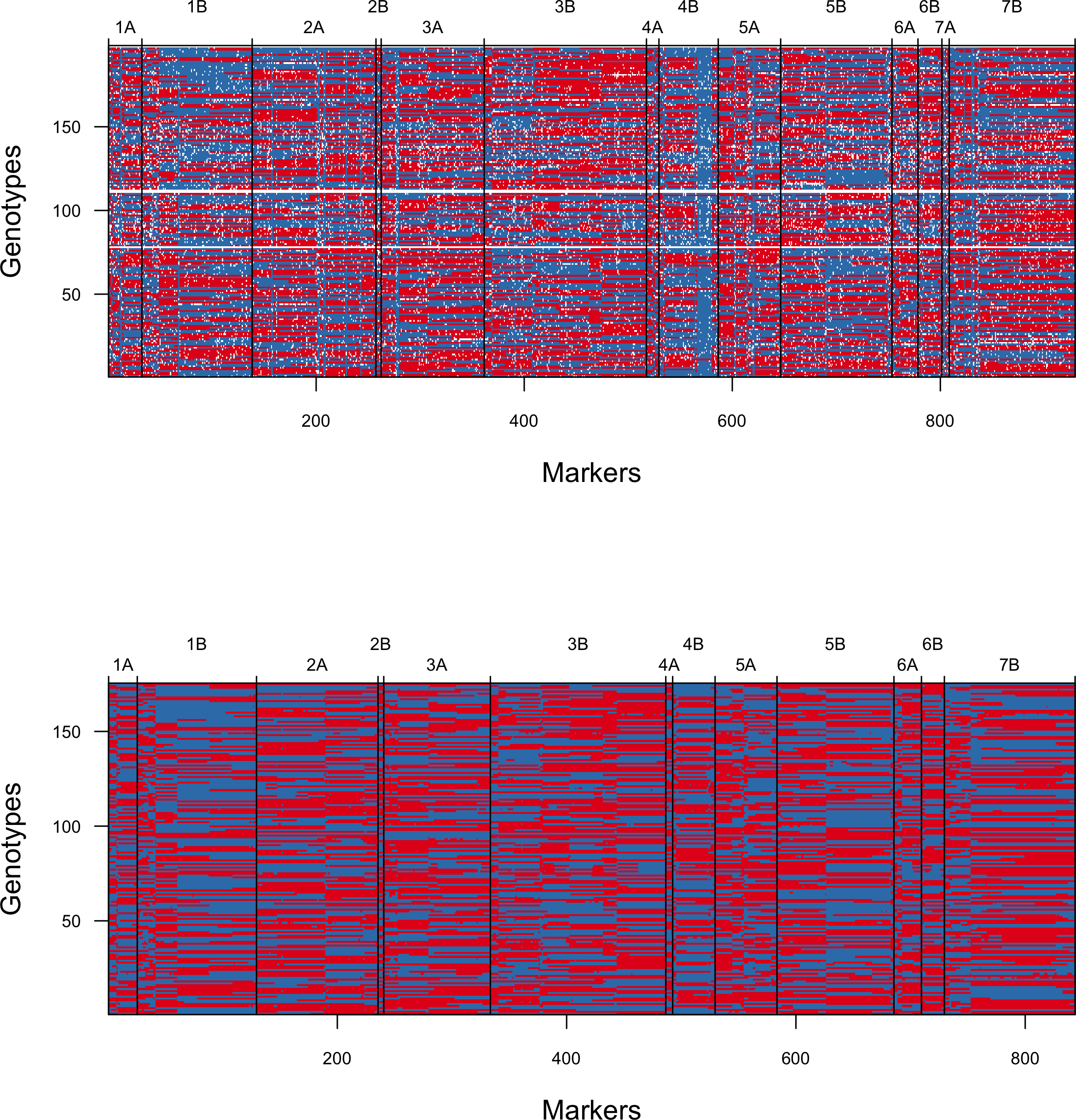

Supplement: S1 Fig — Red represents the allele from the susceptible parent and blue represents the allele from the resistant parent. The white spaces in S1A Fig were missing data and S1B Fig was after imputation and filtering. R-code adapted from Hussain et al. [52]. (TIFF) [file pone.0273993.s001.tiff]

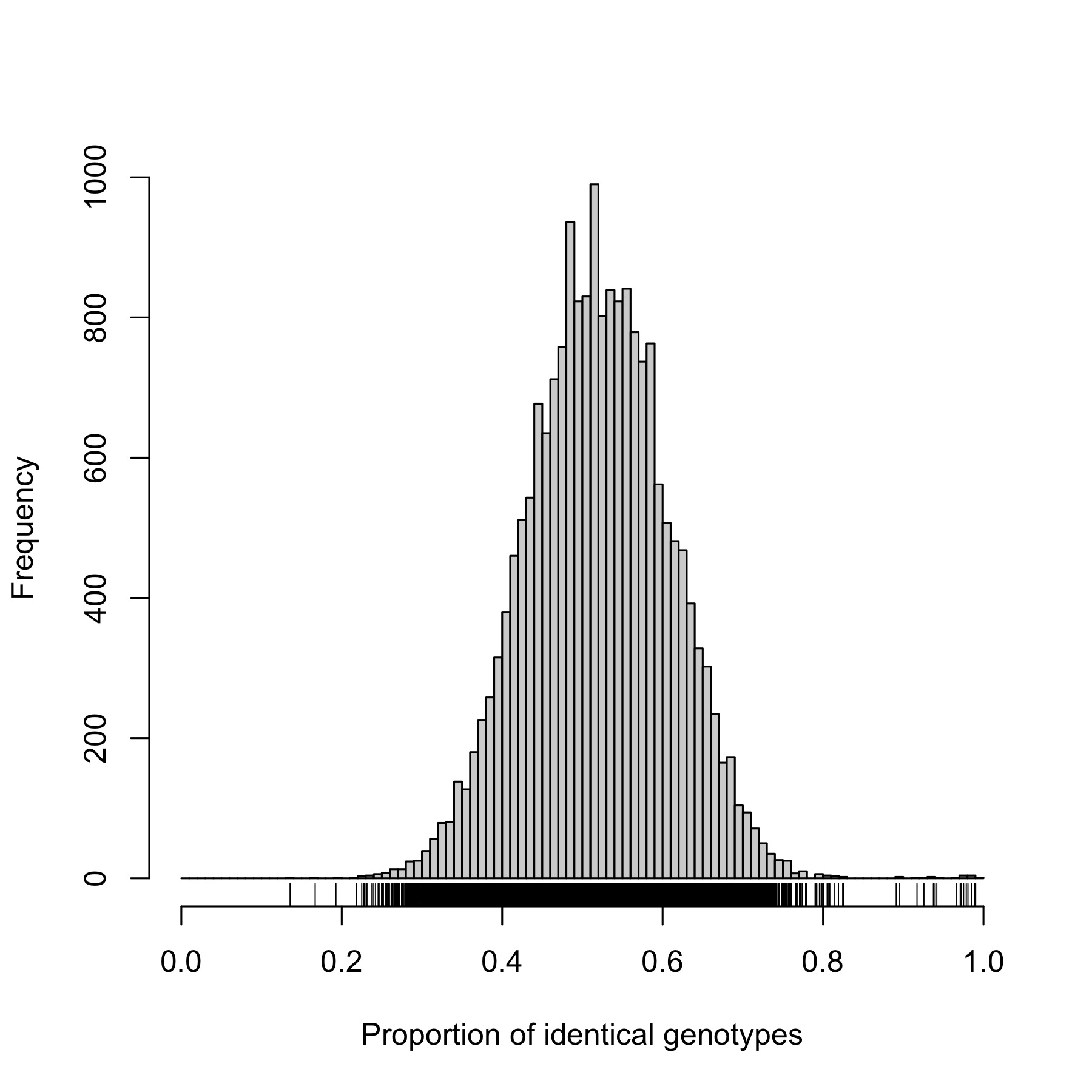

Supplement: S2 Fig — (TIFF) [file pone.0273993.s002.tiff]

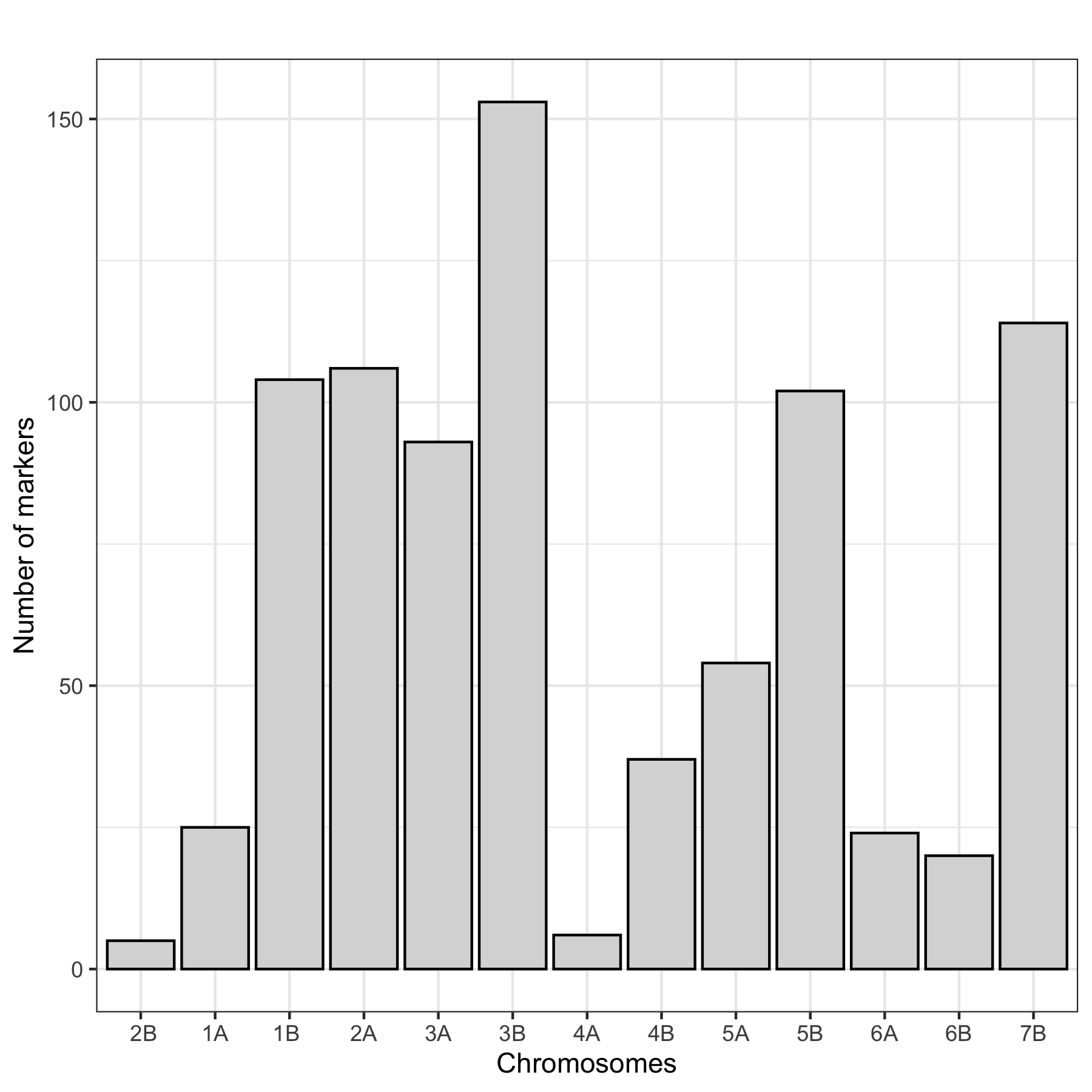

Supplement: S3 Fig — (TIFF) [file pone.0273993.s003.tiff]
