# Supplementary material for: QTL mapping of seedling and field resistance to stem rust in DAKIYE/Reichenbachii durum wheat population
Source: PLoS One. 2022 Oct 6;17(10):e0273993. doi: 10.1371/journal.pone.0273993 (PMC9536579; doi:10.1371/journal.pone.0273993)
Supplement: S1 Table — (DOCX) [file pone.0273993.s004.docx]

| S1Table . Lists of marker genotypes with significant segregation distortion at Bonefferroni threshold. | | | | | |
| --- | --- | --- | --- | --- | --- |
| Marker name | Chr. | No. missing | Genotype | | *P.value* |
|  |  |  | AA | BB |  |
| S1A_539936987 | 1A | 0 | 9 | 169 | 3.89E-33 |
| S1A_540368269 | 1A | 0 | 14 | 164 | 2.51E-29 |
| S1B_647549343 | 1B | 0 | 9 | 169 | 3.89E-33 |
| S1B_653826081 | 1B | 0 | 60 | 118 | 1.38E-05 |
| S2A_9011801 | 2A | 0 | 16 | 162 | 7.17E-28 |
| S2A_10258583 | 2A | 0 | 14 | 164 | 2.51E-29 |
| S2A_58306939 | 2A | 0 | 14 | 164 | 2.51E-29 |
| S2A_58658896 | 2A | 0 | 14 | 164 | 2.51E-29 |
| S2A_73264522 | 2A | 0 | 11 | 167 | 1.39E-31 |
| S2A_73264854 | 2A | 0 | 10 | 168 | 2.35E-32 |
| S2A_79984294 | 2A | 0 | 11 | 167 | 1.39E-31 |
| S2A_124355541 | 2A | 0 | 18 | 160 | 1.87E-26 |
| S2A_479705642 | 2A | 0 | 23 | 155 | 4.43E-23 |
| S3A_629510529 | 3A | 0 | 21 | 157 | 2.12E-24 |
| S3A_630327049 | 3A | 0 | 12 | 166 | 8.03E-31 |
| S3A_633327219 | 3A | 0 | 12 | 166 | 8.03E-31 |
| S3A_729311968 | 3A | 0 | 28 | 150 | 6.00E-20 |
| S3B_213748627 | 3B | 0 | 20 | 158 | 4.48E-25 |
| S4B_16858295 | 4B | 0 | 51 | 127 | 1.22E-08 |
| S4B_50227616 | 4B | 0 | 42 | 136 | 1.85E-12 |
| S4B_583648902 | 4B | 0 | 10 | 168 | 2.35E-32 |
| S4B_587234956 | 4B | 0 | 9 | 169 | 3.89E-33 |
| S4B_587874996 | 4B | 0 | 7 | 171 | 9.96E-35 |
| S4B_592875786 | 4B | 0 | 7 | 171 | 9.96E-35 |
| S4B_593153840 | 4B | 0 | 7 | 171 | 9.96E-35 |
| S4B_594781678 | 4B | 0 | 7 | 171 | 9.96E-35 |
| S4B_599284312 | 4B | 0 | 7 | 171 | 9.96E-35 |
| S4B_600154291 | 4B | 0 | 7 | 171 | 9.96E-35 |
| S4B_600154369 | 4B | 0 | 7 | 171 | 9.96E-35 |
| S4B_603200542 | 4B | 0 | 7 | 171 | 9.96E-35 |
| S4B_604213919 | 4B | 0 | 7 | 171 | 9.96E-35 |
| S4B_604266753 | 4B | 0 | 7 | 171 | 9.96E-35 |
| S4B_605608964 | 4B | 0 | 7 | 171 | 9.96E-35 |
| S4B_605667151 | 4B | 0 | 7 | 171 | 9.96E-35 |
| S4B_605804494 | 4B | 0 | 11 | 167 | 1.39E-31 |
| S5A_222801444 | 5A | 0 | 18 | 160 | 1.87E-26 |
| S5A_639651884 | 5A | 0 | 12 | 166 | 8.03E-31 |
| S5A_639781876 | 5A | 0 | 12 | 166 | 8.03E-31 |
| S5B_457902655 | 5B | 0 | 18 | 160 | 1.87E-26 |
| S5B_660296496 | 5B | 0 | 32 | 146 | 1.29E-17 |
| S6B_655908347 | 6B | 0 | 25 | 153 | 8.47E-22 |
| S7A_631803925 | 7A | 0 | 14 | 164 | 2.51E-29 |
| S7B_47265059 | 7B | 0 | 15 | 163 | 1.36E-28 |
| S7B_59128773 | 7B | 0 | 10 | 168 | 2.35E-32 |
| S7B_60598849 | 7B | 0 | 9 | 169 | 3.89E-33 |
| S7B_60600901 | 7B | 0 | 12 | 166 | 8.03E-31 |
| S7B_221204263 | 7B | 0 | 13 | 165 | 4.54E-30 |
