# Supplementary material for: QTL mapping of seedling and field resistance to stem rust in DAKIYE/Reichenbachii durum wheat population
Source: PLoS One. 2022 Oct 6;17(10):e0273993. doi: 10.1371/journal.pone.0273993 (PMC9536579; doi:10.1371/journal.pone.0273993)
Supplement: S2 Table — (DOCX) [file pone.0273993.s005.docx]

| S2 Table. Summary of analysis of variance and QTL by environment interaction | | | | | | |
| --- | --- | --- | --- | --- | --- | --- |
| QTL name | Source of variation | Df^a^ | Sum Sq | Mean Sq | F Value | Pr( >F) |
| *QSr.cnl-3B* |  |  |  |  |  |  |
|  | QTL | 1 | 14663 | 14662.9 | 40.8052 | 3.103e-10 *** |
|  | ENVb | 3 | 1919 | 639.7 | 1.7802 | 0.1496 |
|  | QTL*ENV | 3 | 8006 | 2668.8 | 7.4269 | 6.705e-05 *** |
|  | Residual | 686 | 246507 | 359.3 |  |  |
| *QSr.cnl-4B* |  |  |  |  |  |  |
|  | QTL | 1 | 2164 | 2164.11 | 5.6097 | 0.01814 * |
|  | ENV | 3 | 1923 | 641.11 | 1.6619 | 0.17392 |
|  | QTL*ENV | 3 | 2364 | 787.85 | 2.0422 | 0.10666 |
|  | Residual | 686 | 264644 | 385.78 |  |  |
| *QSr.cnl-7B* |  |  |  |  |  |  |
|  | QTL | 1 | 10370 | 10370 | 28.2372 | 1.453e-07*** |
|  | ENV | 3 | 1917 | 638.9 | 1.7396 | 0.1575317 |
|  | QTL*ENV | 3 | 6877 | 2292.2 | 6.2416 | 0.0003489 *** |
|  | Residual | 686 | 251932 | 367.2 |  |  |
| ^a^Degree of freedom  ^b^Environment | | | | | | |
