# Supplementary material for: QTL mapping of seedling and field resistance to stem rust in DAKIYE/Reichenbachii durum wheat population
Source: PLoS One. 2022 Oct 6;17(10):e0273993. doi: 10.1371/journal.pone.0273993 (PMC9536579; doi:10.1371/journal.pone.0273993)
Supplement: S3 Table — (DOCX) [file pone.0273993.s006.docx]

| S3 Table. Summary additive effect of QTL identified for seedling and field response to stem rust. | | |
| --- | --- | --- |
| QTL name | Race/ Trial name | Additive effect |
| *QSr.cnl-3B* | JRCQC | -1.15 |
|  | TTRTF | -0.80 |
|  | ETMS19 | -0.27 |
|  | KNMS19 | -7.85 |
| *QSr.cnl-4B* | ETOS19 | -2.75 |
| *QSr.cnl-7B* | KNMS20 | -8.64 |
